# Rationale and study design of the prospective, longitudinal, observational cohort study “rISk strAtification in end-stage renal disease” (ISAR) study

**DOI:** 10.1186/s12882-016-0374-8

**Published:** 2016-10-26

**Authors:** Christoph Schmaderer, Susanne Tholen, Anna-Lena Hasenau, Christine Hauser, Yana Suttmann, Siegfried Wassertheurer, Christopher C. Mayer, Axel Bauer, Kostantinos D. Rizas, Stephan Kemmner, Konstantin Kotliar, Bernhard Haller, Johannes Mann, Lutz Renders, Uwe Heemann, Marcus Baumann

**Affiliations:** 1 Department of Nephrology, Klinikum rechts der Isar, Technische Universität München, Ismaninger Straße 22, 81675 Munich, Germany; 2 Health & Environment Department, AIT Austrian Institute of Technology GmbH, Biomedical Systems, Donau-City-Str. 1, 1220 Vienna, Austria; 3 Medizinische Klinik und Poliklinik I, Department of Cardiology, Klinikum Großhadern, Ludwig-Maximilians-Universität München, Marchioninistr. 15, 81377 Munich, Germany; 4 FH Aachen, University of applied sciences, Heinrich-Mussmann-Str. 1, 52428 Jülich, Germany; 5 Institute of medical statistics and epidemiology (IMSE), Klinikum rechts der Isar, Technische Universität München, Ismaninger Straße 22, 81675 Munich, Germany; 6 Städtisches Klinikum Schwabing, KFH Dialysezentrum Schwabing, Kölner Platz 1, 80804 Munich, Germany

**Keywords:** End stage renal disease, Pulse wave velocity (PWV), Pulse wave analysis (PWA), Montreal Cognitive Assessment (MoCA), Risk stratification, ISAR, Ambulatory blood pressure monitoring (ABPM), retinal vessel analysis (RVA), Hemodialysis, Dialysis

## Abstract

**Background:**

The ISAR study is a prospective, longitudinal, observational cohort study to improve the cardiovascular risk stratification in endstage renal disease (ESRD). The major goal is to characterize the cardiovascular phenotype of the study subjects, namely alterations in micro- and macrocirculation and to determine autonomic function.

**Methods/design:**

We intend to recruit 500 prevalent dialysis patients in 17 centers in Munich and the surrounding area. Baseline examinations include: (1) biochemistry, (2) 24-h Holter Electrocardiography (ECG) recordings, (3) 24-h ambulatory blood pressure measurement (ABPM), (4) 24 h pulse wave analysis (PWA) and pulse wave velocity (PWV), (5) retinal vessel analysis (RVA) and (6) neurocognitive testing. After 24 months biochemistry and determination of single PWA, single PWV and neurocognitive testing are repeated. Patients will be followed up to 6 years for (1) hospitalizations, (2) cardiovascular and (3) non-cardiovascular events and (4) cardiovascular and (5) all-cause mortality.

**Discussion/conclusion:**

We aim to create a complex dataset to answer questions about the insufficiently understood pathophysiology leading to excessively high cardiovascular and non-cardiovascular mortality in dialysis patients. Finally we hope to improve cardiovascular risk stratification in comparison to the use of classical and non-classical (dialysis-associated) risk factors and other models of risk stratification in ESRD patients by building a multivariable Cox-Regression model using a combination of the parameters measured in the study.

**Clinical trials identifier:**

ClinicalTrials.gov NCT01152892 (June 28, 2010)

## Background

Chronic kidney disease, in particular ESRD is associated with a very high cardiovascular morbidity and mortality [1]. As access to dialysis is getting easier and the spectrum of patients suitable for dialysis has broadened, the worldwide use of renal replacement therapy (RRT) is expected to rise significantly in the next years [2]. The life expectancy of ESRD is similar to metastatic cancer disease. For example the overall life expectancy of a patient after initiation of dialysis is 8 years in the age group of 40 to 44 years, compared to 30 to 40 years of life expectancy in the general population [3]. Despite recent advances in the field of dialysis technique [4] and insights into the complex pathophysiology [5] of the changes in ESRD patients, we are far from understanding the disease and even further from having a cure. To understand the complex processes happening in ESRD, we need a comprehensive characterization of the cardio- and cerebrovascular system as the major events and causes of mortality are of cardio-/cerebrovascular nature. Based on these findings we hope to gain a better insight into the pathophysiological processes happening in ESRD and to identify new risk factors and improve risk stratification. With introducing the ISAR study we try to shape the most complex cardiovascular image of the patient, which can be derived from easy and inexpensive measurements. Thus, the methods used in this study could be easily adapted for routine use in dialysis centers worldwide. Overall, we want to establish a cohort that reflects the current medium age of dialysis patients in western countries, as we know that dialysis patients get older. Previous studies included patients roughly 10 years younger than the patients we treat today and no special consideration of particular habitats were reported. Here we report the rationale and the study design of the ISAR study. We describe (1) cross-sectional and prospective study objectives, (2) the methods that will be used in the study and (3) the rationale for choosing these methods.

### Study objectives

Therefore, we aim to (1) record a cross-sectional and longitudinal picture of the vascular bed to identify and follow changes in the vasculature and (2) to improve cardiovascular (CV) and non-cardiovascular (non-CV) risk stratification by following the patients for up to 72 months and recording events. We intend to establish an end stage renal disease cohort of more than 500 patients in Munich city and surrounding area (urban vs. rural) to get an idea of the dialysis patients treated today in Germany which should be comparable with large parts of Europe.

The main goals of the ISAR study are:

1. A cardio/-cerebrovascular characterization of a representative ESRD cohort (cross-sectional and longitudinal)
2. To find associations between novel and established risk factors and cardiovascular disease to get a better mechanistic insight into the pathophysiology of cardiovascular and infectious disease in dialysis patients
3. To follow cardiovascular changes over time (arterial stiffness, central hemodynamics, cardiovascular events)
4. To find new and established risk factors for the prediction of morbidity and mortality (cardiovascular, non-cardiovascular (dialysis-associated))
5. To find associations between neurocognitive scores/testing and end organ damage, cardiovascular risk factors and vascular parameters
6. To establish a serum sample database for the discovery of novel biomarkers in ESRD
7. To develop a robust model to predict cardiovascular morbidity and mortality and death of all causes in a multivariable Cox-regression analysis

We focus on the above mentioned objectives as we have identified them as improvable areas of interest in previous landmark studies, because the problem has either (1) not been addressed before, (2) the population was too small or (3) the baseline characteristics/epidemiology of the population has changed over time, so it is not representative for the actual population of dialysis patients taken care of in dialysis centers in western countries any longer. Furthermore, we seek to develop a combined model of the different risk stratification methods (ABPM, PWA, PWV, autonomous dysfunction, RVA) to improve prediction of cardiovascular and non-cardiovascular events.

In the following, we give an overview of research questions that we want to address after establishment of the planned dataset.

### Ambulatory and office/dialysis blood pressure monitoring (ABPM)

Objectives with cross-sectional results

1. Build a 24-h ABPM reference database for a well-defined dialysis cohort of prevalent dialysis patients that are comparable to the current dialysis population treated in European countries
2. Evaluate the incidence of masked, white coat hypertension and dialysis treatment associated hypertension by comparison of pre- and post-dialysis BP measurements with ABPM recordings
3. Evaluate the frequency of nocturnal hypertension
4. Find associations between 24-h ABPM measurements and target organ damage
5. Find associations between 24-h ABPM measurements and other cardiovascular parameters e.g. retinal vessel diameter, autonomic dysfunction, central hemodynamics and cardiovascular risk factors
6. Evaluate the adherence of dialysis patients to the prescription of 24-h ABPM (% unwillingness to participate in ABPM, time of measurement, number of canceled examinations, time to cancelation)

Objectives with longitudinal results

1. Does 24-h ABPM predict
  - All-cause mortality
  - Cardiovascular mortality
  - Death from infection
  - Hospitalizations due to
    i. Cardiovascular events
    ii. Infectious complications
    iii. Other reasons
2. Compare the predictive value of intradialytic BP measurements with 24-h ABPM

### Pulse wave analysis

Objectives with cross-sectional results

1. Establish a reference database for end stage renal disease patients in terms of central blood pressure values
2. Correlate measures of central hemodynamics with end organ damage
3. Find associations between cardiovascular risk factors and measures of central hemodynamics
4. Find associations between volume depletion within the dialysis session and changes in central hemodynamics

Objectives with longitudinal results

1. Does 24-h PWA predict
  - All-cause mortality
  - Cardiovascular mortality
  - Death from infection
  - Hospitalizations due to
    i. Cardiovascular events
    ii. Infectious complications
    iii. Other reasons
2. Compare the predictive value of intradialytic PWA measurements with 24-h PWA
3. Evaluate longitudinal changes in PWA

### Pulse wave velocity (derived mathematically from PWA measurements)

Objectives with cross-sectional results

1. Establish a reference database for PWV in an end stage renal disease cohort
2. Correlate PWV with end organ damage
3. Find associations between PWV and other cardiovascular and functional variables (e.g. retinal vessel parameters, cardiovascular risk factors, inflammatory biomarkers, neurocognitive score)
4. Associate PWV with end organ damage

Objectives with longitudinal results

1. Does 24-h PWV predict
  - All-cause mortality
  - Cardiovascular mortality
  - Death from infection
  - Hospitalizations due to
    i. Cardiovascular events
    ii. Infectious complications
    iii. Other reasons
2. Compare the predictive value of intradialytic PWA measurements with 24-h PWV
3. Evaluate the progression of arterial stiffness overtime

### Static and dynamic retinal vessel analysis (RVA)

Objectives with cross-sectional results

1. Evaluate the feasibility of this method in dialysis centers and a dialysis cohort (% inclusion, technical feasibility…)
2. Establish a reference database for static and retinal vessel parameters in an end stage renal disease cohort and compare these with normal controls (controls already available for analysis)
3. Correlate RVA parameters (static and dynamic) with end organ damage
4. Find associations between static and dynamic retinal vessel variables and other cardiovascular and functional variables (cardiovascular risk factors, inflammatory biomarkers, neurocognitive score, central hemodynamics)

Objectives with longitudinal results

1. Does static or dynamic RVA predict
  - All-cause mortality
  - Cardiovascular mortality
  - Death from infection
  - Hospitalizations due to
    i. Cardiovascular events
    ii. Infectious complications
    iii. Other reasons

### Electrocardiogram (ECG)

Objectives with cross-sectional results

1. Evaluate the feasibility of this method in dialysis centers and a dialysis cohort (% inclusion, technical feasibility, % of data sets reaching the quality to be included in the analysis)
2. Calculate the prevalence of atrial fibrillation (first detected, paroxysmal, persistent, permanent) over 24 h
3. Determination of autonomic dysfunction with linear and non-linear methods and association of these parameters with end organ damage and cardiovascular risk factors
4. Evaluate the data set for new determinants of cardiac autonomic dysfunction
5. Calculate ECG parameters of hypertrophy and associate with end organ damage
6. Evaluate for arrhythmias other than atrial fibrillation (ventricular tachycardia, ventricular extra systole, supraventricular extra systole)
7. Evaluate basic ECG parameters (QRS duration, QT time, PR time, resting heart rate)
8. Find associations between volume depletion within the dialysis session and changes in heart rhythm (arrhythmias including atrial fibrillation)

Objectives with longitudinal results

Does autonomic dysfunction at baseline predict

1. All-cause mortality
2. Cardiovascular mortality
3. Death from infection
4. Hospitalizations due to
  - Cardiovascular events
  - Infectious complications
  - Other reasons

### Neurocognitive testing

Objectives with cross-sectional results

1. Evaluate the feasibility of neurocognitive testing in a dialysis setting
2. Evaluate the prevalence of cognitive impairment and dementia
3. Associate neurocognitive scores with end organ damage and cardiovascular risk factors
4. Associate vascular parameters (retinal vessel analysis for microcirculation, PWA and PWV for large vessels) with neurocognitive scores

Objectives with longitudinal results

1. Does cognitive impairment or dementia at baseline predict
  - All-cause mortality
  - Cardiovascular mortality
  - Death from infection
  - Hospitalizations due to
    i. Cardiovascular events
    ii. Infectious complications
    iii. Other reasons
2. Evaluate the progression of neurocognitive decline

### Cardiovascular risk stratification

After evaluation of the above data sets and risk stratification by single estimators we aim at building a multivariable Cox-Regression model which will include up to 15 variables depending on the number of cardiovascular events recorded throughout the observation period and the true number of recruited patients (see section on sample size and statistical analysis).

## Methods and design

Substantial requirements for the selection of technical examinations of the cardiovascular system were that they had to be (1) well validated, (2) feasible in daily practice and (3) cost efficient to allow use in a broad set of patients. What methods did fulfill the above mentioned criteria? The steering committee decided to use (1) 24-h Holter ECG, (2) 24-h ABPM and (3) retinal vessel analysis (RVA), as these methods give an excellent estimate of the situation in the macro- and microcirculation and all methods have been very well validated in the last years. As functional parameter of cerebrovascular microcirculation we aim to measure in parts of the cohort the neurocognitive status by Montreal Cognitive Assessment (MoCA) and Clinical Dementia Rating (CDR).

### Study design

The ISAR study is planned as a prospective, strictly observational cohort study conducted in dialysis centers in Munich and the greater Munich area (17 study sites). The Ethics Committee of the Klinikum rechts der Isar of the Technical University Munich and the ethics Committee of the Bavarian State Board of Physicians approved the study protocol. Informed consent is obtained from all patients. An overview of the examinations and follow up appointments can be found in Table 1.

**Table 1:** Schedule of measurements and activities by visit for the ISAR study

	Baseline examination	Follow-up (FU) visits	
	T O (inclusion in the study)	T 24 months	T 6y
Interviewer/Questionaires			
Informed consent	X		
contact information	X	X	X
sociodemographic	X	X	X
Education and employment	X	X	X
Vascular access	X	X	X
Residual kidney function	X	X	X
Dialysis prescription	X	X	X
medical history	X	X	X
cardiovascular risk factors	X	X	X
Family history of CVD	X	X	X
history of prior transplantations	X	X	X
Physical activity	X	X	X
MOCA	X	X	X
CDR	X	X	X
Restless legs score	X	X	X
Oral medications	X	X	X
Examination			
Height, weight, BMI, upper arm circumference	X	X	X
Blood pressure	X	X	X
Procedures			
12-lead holter ECG (24h)	X		
24-h holter blood pressure	X		
Pulse wave velocity (single measurement)		X	X
Pulse wave analysis (single measurement)		X	X
Pulse wave velocity (24h)	X		
Pulse wave analysis (24h)	X		
Retinal vessel analysis (static examination)	X		
Retinal vessel analysis (dynamic examination)	X		
Blood tests (prior to dialysis, fasting), midweek dialysis session			
Serum blood sampling (frozen at −80 °C)	X	X	X
EDTA blood sampling (frozen at −80°)	X	X	X
Clinical follow up			
Hospitalizations (cardiovascular, infectious…)		X	X
cardiovascular events		X	X
cardiovascular mortality		X	X
total mortality		X	X
dialysis routine blood work (quarterly)			
Glucose, albumin	X	X	X
complete blood count	X	X	X
Electrolytes (Na, K, Mg, Ca, Phos)	X	X	X
Blood gas analysis	X	X	X
intact PTH	X	X	X
Iron status	X	X	X
Kt/V	X	X	X
Dialysis treatments			
Dry weight	X	X	X
dialysis prescription (duration, frequency, dialyzer)	X	X	X
Pre- and Post weights	X	X	X
Dialysis flow rates	X	X	X
Blood flow rates	X	X	X
intravenous medications within dialysis session	X	X	X

### Blood specimen collection

All blood samples (serum and EDTA) are taken before a midweek dialysis session. Before taking blood samples, patients are screened for clinical infection, and those with active infection are examined at a later time point. Blood is sampled in serum and EDTA tubes. Serum tubes are centrifuged. Serum is split into aliquots and frozen at −80 °C. EDTA samples are frozen without further processing allowing later DNA extraction. Routine serum chemistry examinations are performed in ISO certified laboratories in the different centers.

### Screening and eligibility

All patients are reviewed by an ISAR study investigator in the dialysis center for the following inclusion and exclusion criteria:

Inclusion criteria

1. age ≥18 years
2. dialysis vintage ≥90 days
3. willingness to participate in at least one technical examination (either ABPM, 24 hour Holter ECG or RVA)
4. written and informed consent.

We plan to recruit a control arm with patients not willing to participate in any technical examination to control for a bias in patient selection, in terms of the possibility that healthier and younger patients are more eager to participate in time consuming examinations.

Exclusion criteria

1. ongoing infection
2. pregnancy
3. malignant disease having a life expectancy of less than 24 months
4. lack of written and informed consent.

### Study procedures/measurements

Measurements are performed in incident dialysis patients recruited in one center after the other. The study subjects examinations are performed in the following order: 1) informed consent, 2) measurement of basic clinical data after review of the patient charts, 3) blood samples are taken before the midweek dialysis session, 4) start of 24-h ECG and 24-h ABPM shortly before a midweek dialysis session, 5) retinal vessel analysis before a midweek dialysis session 6) neurocognitive testing before dialysis in a separate room [6]. The study measurements are performed in each center by the same observers. All observers are trained intensively before patient examination.

#### ABPM and pulse wave analysis

The 24-h ABPM and parameters of arterial wall stiffness or their surrogates (e.g., augmentation index (Aix)) are both recorded in the same time period (24 h) using the oscillometric device, Mobil-O-Graph (IEM, Stolberg, Germany) with integrated ARCSolver algorithms (AIT Austrian Institute of Technology, Vienna, Austria) [7, 8]. The Mobil-O-Graph ambulatory BP monitoring device is a very well established and validated device as well for peripheral blood pressure recording [9] and 24-h pulse wave analysis [10–12], also for ESRD patients [13–15]. The classical BP measuring unit in the device has been validated according to the recommendations of the British and European Societies of Hypertension (BHS and ESH) [9, 16]. The method, equipped with a generalized transfer function (GTF) to derive aortic pressure waveforms, is based on brachial readings acquired in the course of the conventional pressure measurement at the diastolic level [7].

Surrogate parameters of central hemodynamics derived by this technique have been validated against the validation gold standard of intra-aortic catheter measurements and have been compared with well-established, non-invasive readings (e.g., tonometry, echocardiography and magnetic resonance imaging [17]) for aortic pressures, wave reflections [8] or aPWV [11]. Clinical usefulness of this method has been demonstrated recently [18]. Furthermore, feasibility [10] and reproducibility [7] of cuff-based Pulse Wave Analysis (PWA) measurements have been reported. The Mobil-O-Graph PWA monitor with integrated ARCSolver algorithms holds approvals from CE, FDA and JPAL.

#### Static and dynamic retinal vessel analysis

##### Measurement with the static retinal vessel analyzer (RVA)

Using a non-mydriatic retinal camera Topcon NW200 (Topcon, Japan), 30° color retinal photographs of each subject were taken and analyzed by means of Visualis and Vessel Map Software (IMEDOS Systems Ltd., Jena, Germany). The diameters of retinal arterioles and venules are measured as described previously [19] and combined into summary indices [20] – the central retinal arteriolar (CRAE) and venular equivalents (CRVE) – which represent the average arteriolar and venular diameters of that eye, respectively. These are additionally expressed as the retinal arteriolar-venular ratio (AVR). The ratio compensates for possible magnification differences between eyes, and an AVR of 1 indicates that arteriolar diameters are, on average, the same as venular diameters in that eye, while a smaller ratio suggests narrower arterioles. AVR < 0.7 has been shown to be associated with microvascular disorder of different kind [21]. This technique has been shown to be highly reproducible [19, 22, 23] and it can be successfully used as a “population screening tool” [21].

##### Measurements with the Dynamic RVA

Twenty minutes after pupil dilation with topical tropicamide drops (Mydriaticum Stulln; Pharma Stulln, Stulln, Germany) continuous simultaneous measurements of retinal vessel diameters of arterial and venous segments are performed in a randomly chosen eye of a subject using a standard protocol of Dynamic Vessel Analyzer (DVA, IMEDOS Systems, Jena, Germany). This protocol consists of 50 s baseline following by 3 cycles consisting of 20 s of flickering light (12.5 Hz, 530 nm), 80 s constant illumination each. Vessel segments of approximately 1 mm in length located in the upper temporal quadrant 1–3 optic disc diameters away from the optic disc edge are assessed.

Properties of DVA and its measurement principles have been previously described in detail [24–26]. The device allows noninvasive online assessment of vessel diameter, depending on times and locations along the vessel. For this purpose, the DVA consists of a retinal camera (450 FF; Carl Zeiss, Jena, Germany), a digital camera for electronic online imaging, and a personal computer for system control, analysis, and recording of the obtained data. Additionally, each measurement is recorded on videotape, which provides a possibility for off-line reassessment of the data. In cases of insufficient data points in the original assessment, additional measurements of retinal arterial reaction can be taken off-line from videotape recordings using DVA.

#### Electrocardiogram (ECG)

The 24-h 12-lead ECG recordings are recorded using the Lifecard CF digital Holter recorder (Delmar Reynolds/Spacelabs, Nuremberg, Germany). ECG and heart rate variability (HRV) analysis are determined by using commercial equipment (Delmar Reynolds/Spacelabs Pathfinder, Nuremberg, Germany) and previously published technologies by use of customized and validated software. An experienced physician blinded to the patient’s clinical status manually reviews and processes all recordings to obtain the sequence of individual R-R intervals together with beat classification (sinus beat, VPC, artifact). Besides, automatic annotation of the ECG is performed for comparison [27]. RR intervals are then further processed. The 24-h ECG recordings are analyzed in terms of traditional parameters, such as QT, QTc, PQ and QRS intervals [28] and left ventricular hypertrophy (LVH) parameters, such as Sokolow-Lyon Index and Cornell Index [29]. For the analysis of the variability of R-R intervals, several HRV parameters are taken. These range from traditional parameters in the time and frequency domain (e.g., standard deviation of the N-N intervals, total power), over further linear (e.g., Poincare indices, the triangular index) to non-linear parameters (e.g., entropy measures, detrended fluctuation analysis) [30–32]. Furthermore, heart rate turbulence (HRT) and deceleration capacity (DC) are calculated according to previously published technologies using established cut-off values. Briefly, HRT quantifies the baroreflex-mediated short-term oscillation of the cycle lengths following ventricular premature complexes [33]. DC quantifies the mean amplitude of all deceleration-related oscillations of heart rate observed in the recording period [34] and is based on a new signal processing algorithm termed phase-rectified signal averaging (PRSA) which is capable of extracting periodic components out of non-stationary, noisy signals [35].

#### Neurocognitive testing

Cognitive function is measured using the Montreal Cognitive Assessment (MoCA) [36]. The MoCA is a short screening instrument for cognitive impairment, which evaluates the domains attention and concentration, memory, orientation, language, visuoconstructional skills, conceptual thinking, calculations and executive functions. Assessment of the latter function is of particular importance, because, apart from memory function, it seems to be impaired early in hemodialysis patients [37, 38]. Cognitive testing is performed under standardized conditions (before dialysis and alone in a separate room), as proposed previously [6].

In addition to MoCA, the Clinical Dementia Rating (CDR) is applied, being the international standard for evaluation of the overall severity of dementia [39]. The assessment includes an interview with an informant or care-giver of the patient, which covers six cognitive and functional domains: memory, orientation, judgment and problem solving, community affairs, home and hobbies and personal care.

### Clinical covariates, endpoints, statistical considerations and data collection

#### Adjudicated baseline comorbidities

Baseline comorbidity is determined by the ISAR Endpoint Committee with two independent reviews and a final review. If a consensus cannot be reached, other members of the committee review the chart and a majority vote of the committee determines the final comorbidity. Comorbidity will be assessed by the original Charlson Comorbidity Index (CCI) [40] and adapted versions of the CCI for ESRD patients [41–43] as far as this data is available from the records at inclusion. Assigned cause of end stage renal disease is obtained from medical record review, kidney biopsy reviews if available and interview with the patient at inclusion.

#### Adjudicated clinical cardiovascular events and outcomes

##### Mortality ascertainment

The observation period for each participant begins at enrollment and continues for 72 months after inclusion. Mortality events are documented from clinic reports, medical records and interviews with the physicians in the dialysis centers.

##### End points

We aim at building a combined dataset to answer questions about the complex and so far only insufficiently understood pathophysiology behind the extreme cardiovascular and non-cardiovascular mortality in dialysis patients. Final goal is the development of a robust model to predict cardiovascular morbidity and mortality and death of all causes in a multivariable Cox-regression analysis. The study end points and serious adverse events are monitored at the time of the follow-up examinations at 24 and 72 months and entered in the study database. Furthermore we will be continuously informed by the dialysis units of the participants death. On notification of a death event we will gather all available clinical information and if in doubt of the correct diagnosis we will contact the next of kin. A panel of three physicians comprising a nephrologist and a cardiologist will independently adjudicate all CV and non CV outcomes based on medical documentation or interview with the local dialysis units. All analyses of end points are based on the following classification by the end-point committee which has to be agreed on by consensus or majority vote.

##### Outcomes

Major cardiovascular events are defined as:

1. Sudden cardiac death (SCD) defined as a sudden pulseless condition out of the hospital (most probably due to arrhythmia). If the event is unwitnessed SCD will be suspected if the patient has been seen stable within the last 24 h preceding the event.
2. fatal or non-fatal myocardial infarction (MI), defined as chest pain, dynamic troponin change, cardiogenic shock and original ECG or ECG report to distinguish ST-elevated MI vs. non-ST-elevated MI.
3. need for coronary revascularization (coronary artery bypass graft/percutaneous coronary intervention(PCI)/percutaneous transluminal coronary angioplasty) or death after cardiovascular procedure/operation (e.g. cardiac valve surgery, PCI)
4. Death or hospitalization due to congestive heart failure (CHF).
5. ischemic stroke (defined as an acute focal neurologic deficit of sudden onset attributed to the occlusion of a cerebral arterial vessel by a thrombus), non ischemic-stroke (due to major bleeding)

Major non-cardiovascular, non-cerebrovascular events are defined as:

1. Death or hospitalization due to infection or sepsis (pneumonia, urogenital infection, gastrointestinal, endocarditis, shunt infection, cutaneous infection, meningitis)
2. Death or hospitalization due to malignancy
3. Death due to chronic lung disease (chronic obstructive pulmonary disesase (COPD), asthma, emphysema, pulmonary hypertension)
4. Gastrointestinal death (bleeding, pancreatitis, malnutrition)
5. Death or hospitalization due to trauma or accident
6. Death or hospitalization due to other causes

##### Adverse events

As the ISAR study is strictly observational, there are no study related adverse events to be expected. All pathological examinations that are found throughout the data acquisition or in the analysis of the data are immediately communicated to the attending physicians.

#### Sample size

It is intended to recruit a number of patients that allows to detect relevant differences in mortality between groups of interest and that provides sufficient information for development of a multivariable predictive model for mortality. With an included number of 500 patients, we expect to observe about 100 deaths within the follow-up period of 2 years, based on an assumed 2-year mortality rate of 20 % in our population of dialysis patients [44]. So the study is sufficiently powered (power of about 80 %) to detect differences in mortality between two balanced groups (*n* = 250 patients per group, if the true difference in 2-year mortality between the groups is 10 percentage points (25 % vs. 15 %). For a comparison of two unbalanced groups with sizes of 100 and 400 individuals, e.g. for comparison of groups with presence and absence of a potential risk factor, a true difference in 2-year mortality of 30 % vs. 17.5 % could be detected with a probability (=power) of about 80 %. These differences seem realistic considering reported figures from recent studies [44, 45].

In order to model the hazard rate using a multivariable Cox regression model, the number of observed events is the “limiting sample size” [46]. Based on various simulation studies, the number of predictors that can be included in a multivariable regression model that is likely to provide reliable results is assumed to lie within “limiting sample size divided by 5″ and “limiting sample size divided by 20” [46–48]. Consequently, with a sample size of 500 patients leading to an expected number of 100 observed events, about 10 to 15 predictor variables can be included in a multivariable Cox regression model, which appears to be an adequate number given possible candidates and previously proposed prediction models for mortality in dialysis patients [44].

#### Statistical analysis

Baseline characteristics are described with mean and standard deviation for normally distributed continuous variables, with median and 1st and 3rd quartile for not normally distributed continuous variables and with number (%) for categorical data. Statistical testing of baseline data is performed with the χ2 test for qualitative variables. Continuous variables are analyzed with ANOVA and consequent t-tests with correction for multiple comparisons for normally distributed data. For not normally distributed data we use Kruskal-Wallis test. Survival probabilities in the whole study population and for relevant patient groups will be estimated using the Kaplan-Meier method. Estimated 2-year survival probabilities and corresponding 95 % confidence intervals will be presented. Group comparisons will be conducted using the logrank test and the association between quantitative measures and risk will be assessed using Cox proportional hazards models. Hazard ratios with 95 % confidence intervals will be shown. For endpoints with competing risks, as e.g. death from infection or death from a cardiovascular event, cumulative incidence functions will be drawn and cause-specific hazard rates will be compared or modelled, respectively [49]. All statistical tests will be performed two-sided on a level of significance of α = 5 %. Due to the explorative character of the study no adjustment for multiple testing will be performed. One main goal of the study is to develop a prognostic model for the 2 year survival probability. Various models with different combinations of covariates will be fit to the data. Assumptions of linearity and additivity will be checked. Prognostic accuracy of the model will be assessed using the C index. Resampling methods (cross-validation, bootstrapping) will be applied to estimate the model performance in external data and to identify the best model for prognosis of patient survival. It is intended to write a more detailed statistical analysis plan for development of the prognostic model before the analysis is performed.

#### Ethical considerations

As this study is strictly observational there are no adverse events to be expected that are related to the study. As soon as the study observers find pathological results while evaluating the study examinations, the responsible doctor in the dialysis center will immediately be informed. Furthermore, 24-h Holter ECG and ABPM results of all study participants will be handed to the responsible doctors. There will be no treatment recommendation from the study investigators. The complete responsibility for the treatment of the ESRD patients is in the hands of the responsible doctors in the dialysis centers.

#### Data management and privacy protection

The concept, design and execution of the study, including all data management and analysis are entirely investigator driven. Statistical and methodological support is provided from the institute of medical statistics and epidemiology of the Technical University of Munich (TUM, IMSE).

## Discussion

Within this study we aim to establish an ESRD cohort which reflects current epidemiology in dialysis centers worldwide. By measuring multiple cardiovascular variables of micro- and macrocirculation we hope to gain further insight into the complex process that finally leads to an extreme cardiovascular morbidity and mortality in these patients. The strength of the study is the chance to combine established risk stratification parameters like PWV, PWA, autonomic dysfunction, biomarkers of vascular calcification and inflammation into a complex model, as there has never been such a wide array of examinations in a single study.

### ABPM and pulse wave analysis

#### Ambulatory and office/dialysis blood pressure monitoring (ABPM)

ABPM is the gold standard for diagnosis of hypertension to identify white coat hypertension, masked hypertension and nocturnal hypertension which has been stated in recent guidelines [50, 51]. In dialysis patients this method is usually only rarely performed (despite hypertension being one of the leading causes of ESRD) because the BP monitoring while the patient is in the dialysis unit (peridialysis blood pressure) is trusted. In many dialysis centers it is common practice to measure only pre and post dialysis values for SBP and DBP. With these values antihypertensive therapy is refined. Without a doubt the use of out of dialysis blood pressure measurement or even better ABPM does shape a much clearer picture of true BP but due to expenses and unwillingness of the patients this examination is not routinely performed. There is clear evidence that there is a u-shaped curve of mortality in relation to systolic blood pressure [52–54]. This non-monotonic relationship has also been recognized for other risk factors like nutrition and lipid metabolism in ESRD patients. As ABPM has not entered any guidelines for the diagnosis of hypertension in dialysis patients and the value of ABPM itself in ESRD is not established and still debated by experts [55–57], we hope to gain further insight in the feasibility and usefulness of ABPM in ESRD patients.

#### Pulse wave analysis/central hemodynamics

Arterial stiffness as a sign of vascular calcification is a hallmark finding in patients with chronic kidney disease and especially end stage renal disease patients [58]. Estimation of vascular calcification can be performed either directly by imaging coronary calcification [59] or indirectly by monitoring arterial stiffness by (1) determining pulse wave velocity [60] by measuring pressure wave at two sites (common carotid and femoral artery) and calculation of the time delay of both pressure curves or by means of (2) pulse wave analysis where the brachial pulse wave is mathematically transferred into an aortic pulse wave which can be used to derive PWV values in m/s [10, 61]. The huge advantage of using the latter method is the chance to use 24-h pulse wave analysis and not only a single measurement [10]. This is especially important as pulse wave curves change with volume shifts during and in the period after the dialysis procedure [13]. This method has been very well validated in the last years not only in normal subjects [10] and cardiovascular risk cohorts [11, 62] but also in CKD [18] and dialysis patients [13]. Another advantage is the user independency of the method which would allow broad application in dialysis centers, because “classical” measurement of PWV needs an experienced examiner and is time consuming. Therefore, PWV measurement has not entered clinical routine use except in specialized academic centers within study protocols.

### Static and dynamic retinal vessel analysis (RVA)

Retinal microvasculature is easily accessible with non-invasive imaging techniques and allows visualization of the systemic microcirculation without the need of, e.g., gluteal subcutaneous fat biopsy [63] or estimation of microalbuminuria which is impossible in dialysis patients without residual renal function. It has been shown that chronic kidney disease (CKD) associates with arteriolar narrowing [64], but so far there is only very limited information on the retinal vessels in dialysis patients [65]. Despite macrovacular disease we know that microvascular disease contributes significantly to the development of renal failure. By acquiring digital images and extracting quantitative values for retinal caliber values of the arteriolar and venous bed we are able to look for associations with traditional and non-traditional risk factors and renal disease. Besides, we get a clearer picture of the complex vascular changes in both micro- and macrocirculation and the interplay between these two closely connected systems.

### Electrocardiogram (ECG)

Cardiovascular disease accounts for 50 % of deaths in dialysis patients [3]. Sudden cardiac death (SCD) is with 50 % the most common cause of cardiovascular death. Other reasons are coronary artery disease (CAD), congestive heart failure (CHF), valvular heart disease and atrial fibrillation (AF). The prevalence of atrial fibrillation has risen in ESRD patients and is associated with higher mortality rates in this subgroup of patients [66]. In recent years analysis of cardiac autonomic dysfunction has helped to improve risk stratification in patients after a myocardial infarction [33–35]. It has also been shown that cardiac autonomic dysfunction is a good predictor for cardiovascular mortality in CKD and ESRD patients [67, 68]. Furthermore, there is the possibility of cross-analysis of other biosignals like pulsewaves along with the ECG signals [69]. Therefore, we added this easy, non-invasive and operator-independent method to the set of technical procedures in our study.

### Neurocognitive testing

The profile of dialysis patients has changed significantly in the last years [2]. Today patients on dialysis are older and show more comorbidities than a decade ago. Undoubtedly, the comorbidity of cognitive impairment has grown as well and health care providers are more and more faced with the treatment of cognitive impairment and also with the decision if patients are still able and willing to adhere to medical prescriptions and the recommended dialysis treatments. The estimated prevalence of cognitive impairment in dialysis patients of age 55 and older (the major age group of dialysis patients) is reported with up to 70 % [70] and therefore we need a better understanding of the pathophysiology behind the cognitive decline, its relation to vascular changes and dialysis and non-dialysis dependent risk factors and its longitudinal time course. Within this study we could associate cognitive impairment scores with the diverse vascular parameters described above and in more detail below. Therefore, we decided to include a subgroup of patients with the simple and in dialysis patients validated Montreal cognitive Assessment (MoCA) Score [36, 71] and Clinical Dementia Rating Scale (CDR) [39] at different time points.

## Conclusion

Within this study we aim to establish an ESRD cohort which reflects current epidemiology in dialysis centers in Germany and might help to get a better understanding of the pathophysiology driving mortality and morbidity in ESRD. We hope to improve risk stratification by establishing a multivariable Cox regression model with a combination of the parameters studied in the cohort. We sought to design a feasible protocol combining examinations which are easy to perform and mainly user-independent to assure unbiased quality of data. The ISAR study has already finished recruiting patients and is in the process of follow up examinations. We are keen to present first data of the baseline examinations after database lock.

## Abbreviations

ABPM: 24-h ambulatory blood pressure measurement
Aix: Augmentation index
AVR: Retinal arteriolar-venular ratio
CCI: Charlson comorbidity index
CDR: Clinical dementia rating
CHF: Congestive heart failure
COPD: Chronic obstructive pulmonary disease
CRAE: Central retinal arteriolar equivalent
CRVE: Central retinal venular equivalent
CV: Cardiovascular
ECG: Electrocardiography
ESRD: Endstage renal disease
GTF: Generalized transfer function
LVH: Left ventricular hypertrophy
MI: Myocardial infarction
MoCA: Montreal cognitive assessment
PCI: Percutaneous coronary intervention
PRSA: Phase-rectified signal averaging
PWA: 24 h pulse wave analysis
PWV: Pulse wave velocity
RRT: Renal replacement therapy
RVA: Retinal vessel analysis
SCD: Sudden cardiac death
